# Hereditary Angioedema and Gastrointestinal Complications: An Extensive Review of the Literature

**DOI:** 10.1155/2015/925861

**Published:** 2015-08-03

**Authors:** Napoleon Patel, Lisbet D. Suarez, Sakshi Kapur, Leonard Bielory

**Affiliations:** ^1^Department of Internal Medicine, Atlantic Health System, Overlook Medical Center, 99 Beauvoir Avenue, Summit, NJ 07902, USA; ^2^Division of Allergy and Immunology, Rutgers University Robert Wood Johnson University Hospital, New Brunswick, NJ 07103, USA

## Abstract

Hereditary Angioedema (HAE) is a rare autosomal dominant (AD) disease characterized by deficient (type 1) or nonfunctional (type 2) C1 inhibitor protein. The disorder is associated with episodes of angioedema of the face, larynx, lips, abdomen, or extremities. The angioedema is caused by the activation of the kallikrein-kinin system that leads to the release of vasoactive peptides, followed by edema, which in severe cases can be life threatening. The disease is usually not diagnosed until late adolescence and patients tend to have frequent episodes that can be severely impairing and have a high incidence of morbidity. Gastrointestinal involvement represents up to 80% of clinical presentations that are commonly confused with other gastrointestinal disorders such as appendicitis, cholecystitis, pancreatitis, and ischemic bower. We present a case of an HAE attack presenting as colonic intussusception managed conservatively with a C1 esterase inhibitor. Very few cases have been reported in the literature of HAE presentation in this manner, and there are no reports of any nonsurgical management of these cases.

## 1. Introduction

HAE is a condition presenting as recurrent attacks of angioedema usually without symptoms of pruritus or urticaria. It is an autosomal dominant condition typically presenting in childhood, characterized by nonpitting edema of subcutaneous and mucosal tissues and usually associated with the upper respiratory and gastrointestinal systems [1, 2]. Patients may experience nausea, vomiting, diarrhea, pain syndromes, and laryngeal swelling that may be life threatening [3]. This topic review will focus on the gastrointestinal complications of HAE as a potential area of misdiagnosis leading to surgical morbidity. It has been estimated that 1 in 10,000–50,000 persons is affected by HAE across any ethnic group. Although a recent study from Norway proposed that 1 per 100,000 of the population may be affected, the exact prevalence of the disease is not known [2, 4]. The age of onset of HAE is variable and can present in children less than one year old, with laryngeal attacks developing usually after the age of three and increasing in frequency after puberty [3].

## 2. Case Presentation

A 19-year-old female presented to the Emergency Department (ED) with complaints of abdominal pain. The patient was in her usual state of health when she experienced an acute onset of abdominal pain, localized to the right upper quadrant. The pain was described as cramp-like in character, accompanied by numerous bouts of vomiting and diarrhea, both of which were nonbloody. Her past medical history was significant for low complement C4 performed at the time of diagnosis. The patient's father has a known history of type 1 HAE. Her medications include an intravenous (IV) C1 esterase inhibitor (Cinryze) taken every 3 days for HAE symptom prophylaxis and subcutaneous (SC) icatibant (Firazyr) to be used during an acute attack. The patient was on a clinical trial of Cinryze for prophylaxis as instructed by her allergy specialist. She had experienced similar episodes in the past which resolved with immediate treatment with Cinryze and Firazyr. The patient's symptoms were well controlled on this regimen until 1 month ago when her symptoms increased in frequency to 1 episode a week. She denied any change in her daily activities but did admit increased stress due to college final exams. Within an hour of her current symptom onset the patient used one application of Cinryze and Firazyr, but the pain was unrelenting and she decided to go to the nearest ED. In the ED the patient was found to have an elevated blood pressure of 148/100 mmHg. Her physical exam revealed tenderness in the umbilical and right upper quadrant upon light palpation. There was no guarding, rigidity, or rebound tenderness, and Murphy's sign was not elicited. Her head, neck, chest, extremities, and skin exam did not reveal any significant findings.

**Figure 1:**
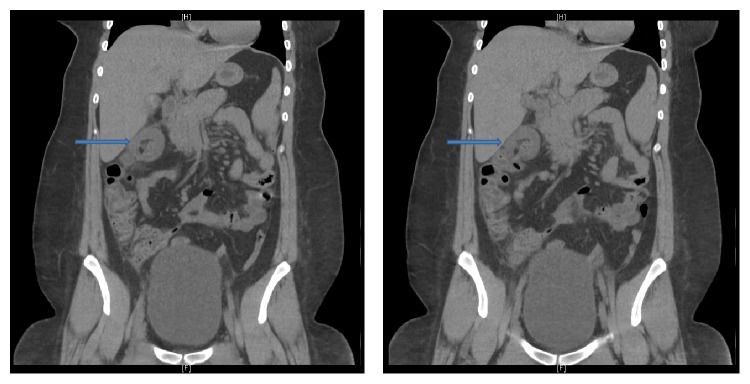
CT abdomen demonstrating colocolic intussusception at the hepatic flexure (arrow).

Laboratory work-up revealed a normal complete blood count and comprehensive metabolic panel. Serum amylase, lipase, and urinalysis were all within normal limits. Her C-reactive peptide was not obtained at the time of evaluation. Abdominal CT scan demonstrated a 2.4 cm segment of colocolic intussusception in the region of the hepatic flexure with a normal appearance of the appendix (Figure 1).

**Figure 2:**
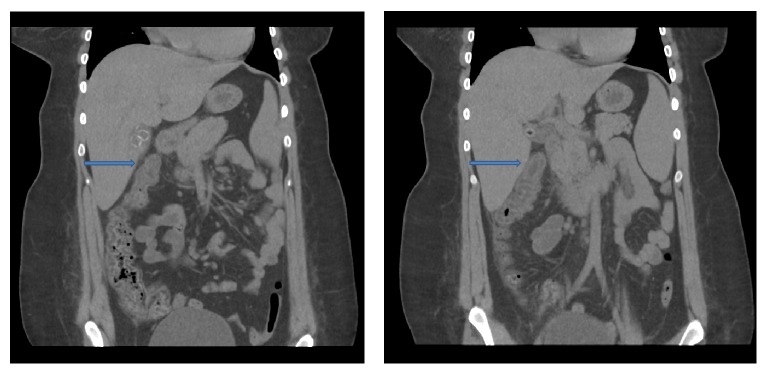
CT abdomen after C1 INH treatment, demonstrating resolution of intussusception (arrow).

The patient was admitted to the hospital and was given supportive care with IV fluids and pain medications and kept nothing per mouth (NPO) while the surgery team was consulted along with her outpatient allergy specialist. Additional diagnosis such as tumor or adhesions causing her abdominal pain was not contemplated given that she had no prior history of abdominal surgeries and the CT findings did not reveal a mass of concern. Her allergist recommended beginning treatment with three 1,000-unit doses of IV Cinryze delivered every 2 hours in an attempt to subside the edema causing the intussusception. The initiation of therapy with IV Cinryze was roughly 4 hours after arrival to the ED. If the treatment failed, then the patient would be scheduled to undergo air-contrast enema for decompression. Overnight the patient's abdominal pain resolved, and repeat CT imaging demonstrated resolution of the intussusception and a normal appearance of the bowel wall with no evidence of obstruction (Figure 2). The patient was able to tolerate advancement in her diet and was discharged home later that day with a follow-up appointment with her allergy specialist.

## 3. Discussion

The angioedema in HAE develops secondary to excess bradykinin production due to low levels of functionally active C1 inhibitor (C1 INH). This leads to the activation of the kallikrein-kinin system causing the release of vasoactive peptides and ultimately angioedema formation [5]. Several types of HAE resulting from a genetic disorder have been identified that are not related to acquired C1 inhibitor deficiency or drug induced angioedema. Type 1 HAE is the cause of the disease in about 85% of HAE patients due to deficiency of the C1 INH protein (quantitative defect). Type 2 HAE comprises the majority of the remaining 15% of patients with HAE with a normal or elevated level of the C1 INH protein but with a functional deficiency (qualitative defect). Both types 1 and 2 are a result of a mutation in the C1 INH gene [3, 5]. A third type of HAE has been found, primarily in women, with normal C1 INH protein and the mutation is actually in the coagulation factor XII gene [5].

Characteristic locations for HAE attacks involve the skin, upper respiratory tract, and gastrointestinal system [1, 2]. Symptoms are self-limited, progressing over hours, and can persist from 1 to 4 days and the frequency of attacks can vary from weekly to a few attacks per year [1, 6]. Premonitory symptoms associated with HAE can develop as little as hours or up to days before the start of an attack [7].

Common prodromal symptoms include nausea abdominal pain, rash, fatigue, muscle aches, numbness, and tingling [8]. Prodromal skin changes can be described as a nonurticarial erythematous discoloration on the extremities and trunk with reticulate and serpentine appearance similar to that of erythema marginatum [9]. Cutaneous attacks of HAE typically involve swelling of the skin, which was present in 97% of episodes in one study with 221 patients. Face, genitals, upper more often than lower extremities, and rarely the neck and trunk were the most notable locations of swelling. Laryngeal edema is the most serious complication that can become life threatening but is a relatively rare event. Only 0.9% of all edema episodes involved laryngeal edema. However, 51% of patients did admit to experiencing some sensation of tightness in the throat, hoarseness, and aphonia/dysphonia in their lifetime. Laryngeal edema can occur alone or with simultaneous swelling of the soft palate, tongue, and uvula [10]. There have also been reports of attacks manifesting as headaches, temporary neurologic deficits, swelling and spasms of the urethra and bladder, joint swelling, chest tightness and pain, and renal colic [10, 11].

Gastrointestinal tract involvement is an important feature and one of the most common in HAE. The difficulty in recognizing gastrointestinal symptoms as being related to HAE often leads to a delay in diagnosis and to unnecessary surgical procedures [1, 12, 13]. The most common symptoms include varying degrees of nausea, vomiting, diarrhea, and abdominal pain, which are the result of intestinal edema [1, 12–14]. The abdominal pain can present acutely or as recurrent pain and is described by patients to be cramping and colicky in nature [14]. The pain patients experience can be moderate to severe in intensity and is usually present in 43–93% of all HAE attacks [12]. Many of these abdominal pain symptoms can occur for many years without any associated respiratory or cutaneous involvement. Not only does the transient edema of the bowel wall cause the aforementioned symptoms, but it may also lead to intestinal pseudoobstruction [13, 14]. The entire gastrointestinal tract can be involved in HAE attacks leading to a wide range of clinical manifestations (Table 1). The oropharynx and esophagus can be involved which leads to feeling of dysphagia. Stomach and small intestinal involvement cause nonspecific findings of abdominal pain, vomiting, and diarrhea. Liver involvement can lead to elevated transaminases, exudative ascites, and reversible parenchymal changes. Pancreatic edema can cause partial duct obstruction, which can present as recurrent episodes of pancreatitis [13, 14]. Constipation was a common finding when there was colonic involvement, with only a few reported cases of intussusception [13–15]. Severe consequences such as circulatory collapse may occur due to a combination of vasodilation, fluid loss from emesis and diarrhea, and fluid extravasation from bowel wall edema and ascites. This can lead to considerable hypovolemia and hemoconcentration. According to one observation study of 33,000 gastrointestinal attacks in 153 HAE patients, circulatory collapse occurred in 4.4% of all attacks [15]. In the same study, there was only one case of intussusception, and the patient underwent surgical resection without reported complications. The majority of abdominal attacks last 2–4 days with preceding symptoms of irritability, fatigue, hunger, aggressiveness, and erythema marginatum [15].

The nonspecific character of HAE symptoms can lead to an extensive work-up and unless there is a high index of clinical suspicion, the diagnosis can be delayed leading to inappropriate surgical interventions. Nationwide surveys in Denmark and Spain found mean delays in diagnosis of 13–16 years, with an international Internet based survey finding of 8.3-year delay in diagnosis [16]. This delay often leads to misdiagnosis and one study of 235 patients found that 1/3 of patients experiencing abdominal symptoms underwent appendectomies and exploratory laparotomies [17].  Table 2 represents key results of the international Internet based survey of HAE patients revealing the unnecessary surgical interventions patients may endure before their disease is identified.

Obtaining a detailed patient history and performing a thorough physical exam are crucial to help direct the medical team to appropriate diagnostic testing. Questions pertaining to time of onset, duration, age at first attack, intervals between attacks, triggering medication and events, family history of similar symptoms, and a thorough review of systems are areas of interest that should be investigated [12]. The physical exam should include skin inspection for cutaneous angioedema, typically nonpitting with no associated pruritus. Tongue, lip, and oral airway swelling may be present as well as stridor on respiratory auscultation. The abdominal exam may be nonspecific and the patient may exhibit diffuse abdominal tenderness on palpation with bowel sounds hyper- or hypoactive and shifting dullness if ascites is present. The physical exam is mostly helpful during the acute attacks [12, 14].

To confirm the diagnosis of HAE it is important to correlate the history and physical exam findings with laboratory and radiographic evidence. The recommended initial screening laboratory testing for HAE includes serum C4 level, C1 INH antigenic protein, C1 INH function/activity, and serum C1q levels which is the result from C1 INH breakdown [3, 14]. The C4 level is typically low in most cases of HAE and is the quickest and most readily available screening test [14, 18]. However, there is one case in the literature of a patient with consistent normal complement C4 levels [19]. The findings of a low C4 along with low C1 INH level and activity and a normal C1q level are confirmatory tests for HAE type 1, while type 2 HAE laboratory findings would reveal a normal C1 INH and C1q levels, but low C1 INH functional activity. Complement studies should be repeated after one month to confirm the results and diagnosis [3, 14]. Most abdominal attacks are not associated with elevations in the white blood cell count, but patients experiencing severe exacerbation may present with elevated neutrophils without bands. An increase in hematocrit was also notable, likely secondary to hemoconcentration from dehydration and fluid translocation to the intestinal wall [13, 20, 21]. A recent study did find a correlation between C-reactive protein levels in HAE patients. Asymptomatic patients with HAE were found to have elevated C-reactive protein levels at baseline. The C-reactive protein levels increased during attacks and were more likely to be elevated in abdominal attacks as compared to other locations [22].  Table 3 lists the most common gastrointestinal disorders and their distinguishing features in comparison with abdominal attacks of HAE.

Radiologic tests may be helpful during initial investigations of abdominal pain episodes but not necessary to confirm a diagnosis of HAE. Abdominal X-ray during an acute attack may show dilated small bowel loops, thickened mucosal folds, air fluid levels, and a “thumbprint” sign representing an area of mucosal edema [14, 23]. An ultrasound of the abdomen can identify ascites and bowel wall edema better than X-ray. Computed tomography (CT) with contrast may be the most sensitive of the three imaging modalities mentioned because of its ability to identify milder degrees of intestinal edema, ascites, and dilated loops of bowel. A CT is also useful in helping to eliminate other potential etiologies for the patient's abdominal pain [14, 23, 24]. Findings on imaging are transient and bowel wall edema and ascites may quickly resolve after the attack subsides and appear normal if studies are delayed [14, 23]. Imaging is generally not required if a patient with a known diagnosis of HAE is having symptoms similar to episodes in the past.

The literature on endoscopic procedures in HAE episodes is minimal, and it is generally not a recommended step during diagnosis. Upper endoscopy reports described the mucosa to appear erythematous and edematous with findings of small nodules and raised erosions [13, 14]. Colonoscopy has revealed areas of extensive mucosal edema leading to almost total occlusion of the colonic lumen, with biopsies showing normal histology [13]. The scarcity of reports about endoscopic evaluation may be due to self-limiting course of HAE and because of the high risk of precipitating an oropharyngeal attack with endoscopic manipulation [13, 14].

The treatment options for HAE patients involve supportive care, individualized action plans, pharmacological treatment, and prophylactic measures. This combination can prevent or minimize future attacks and save the patient from unnecessary exploratory laparotomies, appendectomies, or other invasive procedures. These treatment guidelines are based on the World Allergy Organization (WAO) 2012 and practice parameters developed by a Joint Task Force of American College of Allergy, Asthma and Immunology and American Academy of Allergy, Asthma and Immunology in 2013 [25, 26]. During an acute attack the initial steps in every case should be to assess hemodynamic stability and target therapy to achieve stability. Airway patency and protection should be the first priority because edema in the oropharynx can lead to fatal asphyxiation [14, 25]. Those in severe respiratory distress may need intubation until medical therapy gains levels of efficacy [25, 26]. The patient should have intravenous IV access placed immediately to administer IV hydration to counter the hypotension a patient may develop secondary to fluid shifts and to administer medications [14]. There are 3 medications that are currently approved for treatment in acute attacks in HAE that include a plasma-derived C1 INH for intravenous administration and bradykinin antagonist and inhibitors icatibant and ecallantide via subcutaneous administration [14, 25, 26]. All these first line options have shown themselves to be safe and effective in acute attacks. It is recommended that all patients with HAE should have access to these on-demand therapeutic agents, which the patient may self-administer, as early treatment has been shown to be advantageous [26]. Plasma-derived C1 INH replacement protein marketed as Berinert is made from pooled human blood and works by replacing the deficient protein thereby inhibiting angioedema pathways. The adverse side effects that have been reported include nausea, vomiting, abdominal pain, muscle spasms, diarrhea, headache, and rash [14]. Some thrombotic events have been noted in premature neonates at extremely high doses; however, this has occurred in off-label use [26].

Icatibant marketed as Firazyr is a bradykinin receptor antagonist that has been approved for on-demand use by the FDA in acute attacks. Bradykinin can cause angioedema by activation of B2 bradykinin receptors. This pathway is blocked by icatibant because the medication competitively binds to these B2 receptors. Efficacy studies have shown that when compared to placebo and tranexamic acid significantly more patients had symptom relief at the 4-hour follow-up period with icatibant [27]. Comparing the icatibant treatment group to placebo, initial symptom relief occurred at 0.8 hours compared to 3.5 hours, and complete symptom relief occurred at 8 hours compared to 36 hours. None of the patients treated with icatibant required any additional rescue medications before symptom resolution [28]. It is recommended in patients 18 years or older. Side effects reported include transient local injection site irritation, but no allergic reactions have been reported [26]. Another option is treatment with a kallikrein inhibitor such as ecallantide, marketed as Kalbitor. By inhibiting kallikrein activity, the cleavage of kininogen to bradykinin is inhibited therefore impeding edema progression [26]. It has been approved by the FDA for on-demand treatment in patients 16 and above for all types of HAE attacks [14, 26]. Some patients may develop nonneutralizing antibodies to the drug after repeated uses, leading to anaphylactoid-type reactions in 2-3% of the patients. This is why the FDA recommends that a trained healthcare provider administer the medication, preferably in a facility with the ability to manage anaphylaxis [25]. Another agent approved for the treatment of acute attacks of HAE is conestat alfa (branded as Ruconest), a human recombinant C1 esterase inhibitor purified from the milk of transgenic (genetically modified) rabbits. It is intended to restore the level of functional C1 esterase inhibitor in the plasma, which will subsequently treat the acute attack of swelling. In comparison to the plasma-derived C1 INH, it demonstrated comparable time to first improvement and to resolution of symptoms, making it a reasonable alternative [29].

Epinephrine, antihistamines, and corticosteroids have been proven to be ineffective and are not recommended as part of the HAE treatment regimen. This is because the swelling caused in HAE is due to bradykinin, and the medications mentioned above do not antagonize the generation of effects of bradykinin. Prior to the research and development of the present approved interventions, fresh frozen plasma FFP had been used to abate acute HAE attacks because it contains high circulating levels of C1 INH protein, but it also contains prekallikrein, kininogen, and coagulation factor XII which may lead to worsening of attacks in some patients. Caution is advised if this treatment option is considered [25].

Novel to our case is the fact that after being treated with Cinryze the patient's intussusception resolved completely, as confirmed by CT scan. This management strategy prevented further invasive interventions, including air-contrast enema. There are only a few reports of intussusception in the literature regarding HAE patients, all of which relied on surgical management as the ultimate treatment of this complication, given the lack of evidence on alternative management, along with the pressing factor of worsening complications if surgery is delayed [15].

A necessary part of the treatment regimen is to prevent future attacks. One method can be achieved through an individualized patient action plan. The action plan can be established between the patient and a healthcare professional in order to educate patients on recognizing an attack, recognizing triggers, learning how to self-administer treatment, and planning routes to facilitate access to healthcare. Patients should be advised to carry an identification card to assist healthcare professionals in delivering care [26]. Patients may need short- to long-term prophylaxis if an invasive procedure or stress event is expected. Prophylaxis in the short term can be achieved with C1 INH replacement and short-term therapy with high dose 17 alpha-alkylated androgens with FFP and plasma reserved for those cases where approved medications are not immediately available [14, 25]. The need for long-term prophylaxis must be individualized based on the patient's frequency and severity of attacks. Low to moderate doses of androgens have been effective in long-term prophylaxis because androgens increase the serum levels of C1 INH and reduce the likelihood of attacks [30]. Antifibrinolytics, such as tranexamic acid and epsilon aminocaproic acid, have also been shown to provide long-term prophylaxis but are less effective than androgens [3, 25]. The use of antifibrinolytics is reported to have higher adverse effect profiles such as coagulation defects with increased bleeding and hypercoagulable conditions, so cautious use is recommended when using these agents [3]. Plasma-derived C1 INH has been effective for long-term prophylaxis because of its long plasma half-life. Reductions in frequency, severity, and duration of attacks have been described in double blind placebo controlled studies with Cinryze, a C1 INH concentrate [31]. It has been FDA-approved for adolescent and adult prophylaxis [25].

The prognosis for patients with HAE before current treatment modalities reached as high as 25–50% in some families with the cause of death almost always secondary to laryngeal edema and fatal asphyxiation [32]. A recent study found that mortality was 29% in patients with undiagnosed HAE compared to 3% in patients with a known diagnosis of HAE [33]. This stresses the importance of early diagnosis and that patient education and access to treatment can greatly reduce mortality.

## 4. Conclusion

Gastrointestinal symptoms are a common feature of HAE attacks and can present in a wide array of clinical manifestations. Symptoms can be nonspecific and may overlap with other abdominal conditions leading to delay in diagnosis and treatment. Physicians should consider HAE as a differential diagnosis when presented with a cause of unexplained abdominal pain. A combination of an individualized action plan, pharmacologic therapy, and prophylactic measures can help prevent years of patient distress and unnecessary surgeries and decrease mortality.

## Figures and Tables

**Table 1:** Gastrointestinal manifestations of Hereditary Angioedema.

Site	Clinical manifestation	Frequency (%)
Skin	Swelling and edema	97% [10]

Oropharynx	Laryngeal edema	0.9% [10]
	Tongue swelling	0.3% [10]
	Dysphagia	16% [34]

Abdomen	Nausea and vomiting	88% [34]
	Crampy and colicky abdominal pain	43–93% [12]
	Abdominal distention	72.8% [15]
	Ascites	30% [15] 80% [35]
	Diarrhea	15% [36] 65% [14]

Circulatory system	Hypovolemic shock	4.4% [15]

Less frequent presentation	Pancreatitis	Rare [13, 14, 37]
	Intussusception	Rare [13–15, 38]
	Tetany	Rare [15]
	Dysuria	Rare [39]

**Table 2:** Key findings of an international Internet based survey of HAE patients in relation to surgical interventions.

Population group	Number of patients who underwent unnecessary surgery due to misdiagnosis (*n*)
Patients in the United States with HAE	24 out of 125 (19%)
Patients in the United Kingdom with HAE	12 out of 52 (24%)

**Table 3:** Clinical presentation of HAE abdominal attacks in comparison to other gastrointestinal disorders.

Disorder	Sign/symptom	Laboratory data	Distinguishing features
HAE abdominal attacks	Nausea, vomiting, diarrhea, crampy abdominal pain	Type 1 HAE: low C4 and C1 inhibitor level/activity, normal C1q level [3] Type 2 HAE: normal C1 inhibitor level, low activity, normal C1q level [3]	History of HAE, colonoscopy: massive segmental mucosal edema [13] CT abdomen: intestinal edema, ascites, dilated loops Attacks usually begin in childhood, but they can occur at any age [34]

Acute diverticulitis	Acute LLQ abdominal pain and tenderness, fever, anorexia, nausea, vomiting, constipation or loose stools	Mild to moderate leukocytosis	LLQ palpable abdominal mass CT abdomen: evidence of colonic diverticula, wall thickening, pericolic fat infiltration, abscess formation or extraluminal air or contrast. At age of 40 less than 5% are affected, at age of 60 that number is 30%, and by age of 80 50–65% of adults are affected [40]

Acute appendicitis	Periumbilical abdominal pain that migrates to RLQ with noted rebound tenderness, anorexia, nausea, vomiting, fever	Leukocytosis with neutrophils >70%, elevated levels of CRP, SAA, ProCT [41]	Peak incidence occurs at age 10–19 years [42], Alvarado Score >7 meets criteria for surgical appendectomy CT abdomen: enlarged appendix diameter >6 mm, appendiceal wall >2 mm thick, inflammatory compression of adjoining adipose tissue, RLQ abscess formation, calcified appendicolith [43]

Small bowel obstruction (SBO)	Diffuse abdominal pain, colicky with waxing/waning characteristic, nausea, vomiting, abdominal distention and tenderness, hyperactive or hypoactive bowel sounds, feculent emesis Peritonitis should be suspected when rigidity, rebound tenderness, or guarding presents [44]	Leukocytosis, hemoconcentration, electrolyte imbalance	Most common in adults with history of abdominal surgery raising suspicion for peritoneal adhesions (75% cases); the second most common cause is hernias Plain film of abdomen displays air-fluid levels, small bowel distention and paucity of air in rectal vault [44] Passage of stool and flatus do not rule out SBO [44] CT abdomen with contrast is diagnostic method of choice

Pancreatitis	Acute onset of abdominal pain, located in epigastrium with radiation to back, nausea and vomiting, low grade fever, tachypnea, epigastric tenderness to palpation	Leukocytosis, hemoconcentration with elevated hematocrit, elevated serum amylase and lipase	Most common occurrence in childhood between ages of 15 and 19 years History of gallstones or alcohol abuse CT abdomen with IV contrast is recommended when suspecting pancreatic necrosis, worsening response to therapy, or questionable diagnosis [44]

Inflammatory bowel disease-ulcerative colitis (UC) and Crohn's disease (CD)	UC: bloody diarrhea, with symptoms of urgency and tenesmus [45] CD: chronic or nocturnal abdominal pain, diarrhea Weight loss, fever; rectal bleeding may or may not be present; extraintestinal manifestations including inflammation of eyes, skin, or joints [46]	Elevated acute phase reactants CRP, ESR UC: p-ANCA positive CD: ASCA positive, p-ANCA negative	Both: most frequently diagnosed in the second decade of life. Stool examination to rule out infectious etiology UC: disease limited to colon. Sigmoidoscopy or colonoscopy: loss of vascular pattern, friability and ulceration Biopsy: crypt atrophy, increase presence of lymphocytes and plasma cells at crypt bases [45] CD: primarily involving distal ileum, though any part of alimentary tract may be involved in a transmural inflammatory pattern Endoscopy: deep serpiginous ulcers and “cobblestone” appearance Biopsy: granulomas noted on specimen [47]

Intussusception	Abdominal pain, nausea, vomiting, diarrhea, hematochezia [48]	Similar to bowel obstruction: leukocytosis, hemoconcentration, electrolyte imbalance	Peak age at presentation is 4–8 months History of tumor or prior abdominal surgery CT abdomen: “target sign” indicative of intussusception [49]

Celiac disease	Abdominal discomfort, weight loss, diarrhea, increased flatus	Iron and folate deficiency, steatorrhea, hypoalbuminemia, hypocalcemia, elevated serum transaminases	May manifest as early as childhood after introduction of gluten in diet Positive serologic testing serum IgA anti-tissue transglutaminase and IgA anti-endomysial antibody have sensitivities of 80–95% and specificities of 95–99%. Mucosal intestinal biopsy showing blunted villi, hyperplastic crypts with increased number of mitotic figures [50]

HAE: Hereditary Angioedema, LLQ: left lower quadrant, RLQ: right lower quadrant, CRP: C-reactive protein, ESR: Erythrocyte Sedimentation Rate, SAA: Serum Amyloid A, ProCT: serum procalcitonin, p-ANCA: perinuclear antineutrophil cytoplasmic antibodies, and ASCA: anti-*Saccharomyces cerevisiae*.
